# Nanosystems for modulation of immune responses in periodontal therapy: a mini-review

**DOI:** 10.3389/fdmed.2024.1509775

**Published:** 2025-01-06

**Authors:** Anirudh B. Acharya, Usha Hegde, Swetha Acharya

**Affiliations:** ^1^ Department of Restorative Dentistry, College of Dental Medicine, University of Sharjah, Sharjah, United Arab Emirates; ^2^ Department of Oral Pathology, JSS Dental College and Hospital, JSS Academy of Higher Education and Research (JSSAHER), Mysore, Karnataka, India

**Keywords:** nanotechnology, immunomodulation, nanosystems, nanomedicine, periodontitis, therapy

## Abstract

Periodontitis is one of the most common oral diseases. It is generally treated by non-surgical and/or surgical therapy with adjunctive approaches for prevention and control. The current understanding of the pathogenesis of periodontitis has unraveled the importance of the inflammatory and immune reactions to combat periodontitis whose etiology is an overlap of microbial, genetic, and environmental factors in a susceptible host. Based on this premise, many therapeutic modalities have been investigated or attempted to resolve this inflammatory disease. Amongst these, nanomedicine has been shown to have therapeutic applications in periodontitis, especially focused on immunomodulation because periodontitis is characterized by over-reactive immune response. This mini-review explores the potential of nanosystems in treating periodontitis by providing an overview of the research efforts in this field of therapeutics. The unique physicochemical and targeting properties of nanosystems seem to be potentially effective platforms for treating periodontitis.

## 1 Introduction

The immune system is accountable for the identification and subsequent nullification or elimination of disease-causing entities (1). An aberrant immune system is involved in inflammatory diseases, allergies, and cancers. The host immune response features innate and adaptive immunity (2). The former develops at birth, including the pre-determined components that can instantly protect the host against damaging insults (3). After the physical barriers such as the mucosa/skin are penetrated, several constituently existing soluble mediators and effectors swing into action. An assortment of defense cells (phagocytes and leukocytes) also responds. If the pathological agents are not entirely neutralized by the innate immunity, the adaptive immune responses are triggered into protective action (4). Although the adaptive immune response is relatively slow, it is highly specific and can respond by “memory” in the future (5). This is due to the role of T-, and B- lymphocytes that orchestrate cell-mediated(involving, for example, cytotoxic T-cells) and humoral (involving, for example, production of antibodies) immunity (6). The “immune surveillance” process of the immune system is vital for our protection against diseases (7). Although effective in defending the body against insults ranging from injuries, infections, or cancers, immune cells may also exhibit aberrant behavior/activity leading to uncontrolled inflammatory responses, allergic reactions, and autoimmune diseases (8, 9). This paved the way for research in the modulation of the immune system for therapeutic purposes. Modulation of the immune system has been termed “immunotherapy”, “immunoengineering”, and “therapeutic immunomodulation”, especially in cancer research and therapeutics (10–12). Immunotherapy conceptually functions by controlling the human immune system (via suppression or stimulation) (13). Immunotherapeutic approaches have shown successful and promising results, albeit, with challenges (some immunomodulatory entities may be unfavorably hindered by cytotoxicity, lack of solubility, or decreased bioactive properties) (14, 15).

In the past few decades, nanosystems have been applied to modulate immune behavior for therapeutic advantages. These nano-size immunomodulatory systems can effectively act together with cell surface receptors bringing about cellular/molecular modulation ensuing in therapeutic beneﬁts (16, 17). The use of immunotherapy in inflammatory diseases is being explored. Inflammation is a key aspect that influences a variety of oral diseases, and hence, immunomodulation with nanosystems is being recognized as a potential immunotherapeutic approach (18).

This mini-review provides insights into the potential role of nanosystems in the modulation of immune responses and potential synergistic effects in periodontal therapy.

## 2 Overview of periodontitis

Periodontitis is characterized by gingival inflammation, periodontal pockets, loss of clinical attachment, and alveolar bone resorption, and if untreated, subsequently leads to loss of teeth affecting oral function and esthetics (19). Because of its high prevalence (20, 21), and its established association with diseases such as diabetes mellitus, cardiovascular and respiratory diseases, rheumatoid arthritis, etc. (22), it negatively influences general health and quality of life.

Periodontitis is known to occur after complex interactions that happen between the oral biofilm (dental plaque) and the host immune response, with the latter considered to be a risk for periodontal tissue destruction by 80% (23). It is recognized that the oral microbes contribute to periodontal disease, and mechanical removal of oral biofilm that covers the tooth and tooth-root surface is efficient to reduce periodontitis (24). However, a model of host-microbe interactions in the pathogenesis of periodontitis (25), proposed that although a pathogenic biofilm is a necessary factor for the occurrence of periodontitis it is insufficient alone to be the causative factor of the disease. In the past, pathogenesis was associated with certain organisms found in larger proportions, including *Porphyromonas gingivalis, Treponema denticola,* and *Tannerella forsythia,* triggering an inflammatory reaction (26). However, newer information shows the polymicrobial nature of periodontal health and disease, with the disease being recognized as a dysbiotic state of the periodontal microbiota resulting in an altered host-microbe immune response mediating the local inflammatory processes causing the damage to the periodontium (27). Primarily, these processes try to get rid of invading organisms. However, absence of mechanisms of pro-resolution are believed to be responsible for the acute inflammatory state to become chronic (26–28). Some toxic products that are linked with the microbial shift, including lipopolysaccharides that are present in the gingival sulcus, can have access to the gingival tissue, creating a local inflammatory response marked by neutrophils, lymphocytes and macrophages (29, 30). For the purpose of clearing the insult, pro-inflammatory cytokines, including interleukin (IL)-1β, IL-6 and tumor necrosis factor (TNF)-*α*, are locally produced and is linked to connective tissue destruction resulting from host matrix metalloproteinase activation; however, cytokines including IL-1β, IL-6 and TNF-α, in addition to the arachidonic acid metabolite prostaglandin E2, are strongly related to periodontal diseases’ onset and progression (31).

These inflammatory mediators have been seen to be elevated in areas showing periodontal tissue damage, as they cause bone resorption and induce production of matrix metalloproteinases (like collagenase) (32). Symbiosis is developed in the oral microbial community with the periodontium to establish periodontal health. However, disturbance of this microbial community, either by overgrowth of specific or nonspecific microorganisms or by alterations in the local host response, causes dysbiosis where the oral microbial community may now create a diseased condition, driving periodontal tissue destruction (33). Evidence indicates that it is mainly the host immune response by hyperactive immune cells that causes periodontal tissue destruction, and the variability of the host responses can be attributed to the variability in the clinical manifestations of periodontitis (23, 34).

## 3 Nanosystems

From a historical perspective, in the 1850s Michael Faraday, while studying dispersion effects, developed gold nanoparticles, and after a century Richard Feynman proposed the idea of nanotechnology; in 1974 Norio Taniguchi coined the term “nanotechnology,” with extensive contributions subsequently by K. Eric Drexler to molecular nanotechnology (35). Later, the terms “nanosystems” and “nanomachines” came into use (36, 37). Nanosystems are described as particles with sizes ranging from 1 to 100 nm, or particles that have one dimension that is less than 10 nm (38, 39).

The basis for nano-scale material is that when large materials are reduced to a smaller size (i.e., molecular size) they begin showing new attributes. At this nanoscale, surface properties experience a profound change due to greater chemical reactivity and surface free-energy. There is a higher surface-to-volume ratio that alters the efficiency and toxicity of these nanosystems. Hence, nanosystems have distinctive properties (chemical, biological, and physical) to attain selectivity, specificity, and control of their functionality (40, 41).

From an immunomodulatory perspective, nanosystems are nanostructured materials designed to interact with the immune system in a controlled way. These systems can be engineered to modulate immune responses, either by suppressing excessive inflammation or by enhancing the body's ability to fight infections. Nanosystems have an excellent ability to specifically modulate the immune responses for a therapeutic benefit. The nano-sized property of nanosystems permits them to efficiently interact with immune cells, and consequently undergo endocytosis by hyperactive immune cells in the sites of inflammation. Hence, nanosystems influence the hyperactive immune cells and decrease inflammation. Nanosystems may bring about immunomodulation by acting on the production of cytokines or neutralizing them, infiltrating and modulating/inhibiting hyperactive immune cells, tempering oxidative stress, polarization of macrophages, and stimulating T-cell mediated tolerance (12).

### 3.1 Types of nanosystems

In the context of immunomodulation, nanosystems have been developed for immunostimulation, i.e., activation of antigen presenting cells, T-cells, and, for immunosuppression that involves suppression of macrophages and regulatory T-cells (T-reg cells). These nanosystems can be fabricated using biodegradable and immunologically inert materials, such as polymer, lipid, and carbohydrate nanoparticles, which act as ‘nanocarriers’ for the controlled delivery of immunomodulatory molecules. Alternatively, they can be made using nanomaterials with known immunoactive properties, including metallic nanoparticles {such as carbon nanotubes, gold (Au), zinc (Zn), iron (Fe), silver (Ag), and copper Cu)}. Natural biologically derived materials that elicit immunomodulatory effects, like extracellular vesicle nanoparticles, liposomes, exosomes, enzymes, cell membranes, hyaluronic acid, rosmarinic acid, and bilirubin, have also been explored. These approaches have led to the development of hybrid nanosystems, including biomimetic cell membrane-coated nanoparticles, trigger-responsive nanosystems, hybrid nanogenerators, and cell membrane-coated nanoparticles (9, 12, 42–44).

The nanoscaled dimensions of nanosystems permit them to effectively interact with the surface receptors on the immune cells of interest and final uptake by these immune cells to render the preferred effect (16).

### 3.2 Mechanism of nanosystems

Nanosystems exert their immunomodulatory action through several mechanisms. An exaggerated cytokine (pro-inflammatory cytokines such as IL-1β, IL-6, IL-17, IL-18, TNF-α, and anti-inflammatory cytokines like IL-4, IL-10, interferon [IFN]-*γ*, transforming growth factor [TGF]-β production by hyperactive immune cells is observed in inflammatory diseases, including periodontitis (45, 46). Such an excessive production of pro-inflammatory cytokines results in persisting inflammation causing tissue destruction. A T-helper 1/T-helper 2 cell (Th1/Th2) balance is also necessary to avoid inflammatory tissue destruction (47). Nanosystems are devised to selectively modulate the production of pro- and anti-inflammatory factors by the hyperactive immune cells, i.e., inhibit the pro-inflammatory cytokines production and stimulate anti-inflammatory cytokines production, by regulating gene expression and/or the signaling pathways[bilirubin nanoparticles reduce the production of pro-inflammatory cytokines by suppressing the signaling cascades responsible for cytokine production like nuclear factor-kappa B (NF*κ*B) signaling cascade] to resolve the inflammation (48–50). Cell membrane-coated nanosystems can also block the activity of excessively produced pro-inflammatory cytokines (51). The next type of mechanism is immune cell infiltration modulation. Persistent or chronic inflammation progresses because of the infiltration of hyperactive immune cells into the inflammatory sites (52). Nanosystems are capable of inhibiting this infiltration by blocking/downregulating the chemotactic activity of the hyperactive immune cells (biodegradable nanoparticles have docked onto neutrophils and decreased their infiltrative capacity) (53, 54). Nanosystems can bring about favorable immune responses by macrophage polarization modulation. Macrophages have an important role in the immune reaction during which they are polarized to suppressive type or pro-inflammatory type (55, 56). It has been proposed that nanosystems can help in curbing the macrophage polarization to pro-inflammatory type (M1) and promote polarization to the suppressive type (M2) (57–59). Other mechanisms by which nanosystems aid in beneficial immunomodulation include enhancing the tolerogenic capacity of regulatory T-cells (T-reg) (60, 61), decreasing the numbers of hyperactive immune cells by selective killing (62–64), and scavenging excess reactive oxygen species (ROS) that have been produced during the inflammatory process (65–67).

Several immunomodulatory strategies are being aimed at controlling the inflammatory/immune responses. Nanosystems are biocompatible with exceptional physicochemical properties and are effective as part of drug delivery platforms, and their use in immunomodulatory therapy approaches seems promising (68). The mechanisms of action of these nanosystems have been outlined earlier. Construction of nanosystems for immunomodulation entails considering the composition, size, and surface characteristics (for suitable modification) to engineer them for biodegradation (for example, by using biocompatible materials like polylactic/polyglycolic acid polymers) in the human body (69–72). Bearing these in mind, the distinct characteristics of nanosystems can be potentially utilized in designing them to interact with immune/hyperactive immune cells and tissues of interest for precision and personalized therapy to restore health. Nanosystems containing nanoparticles, polymers, exosomes, lipids, liposomes, can be targeted to reach local sites that obviate systemic side effects (73–76).

## 4 Nanosystems in the treatment of periodontitis

Periodontitis is usually treated primarily by non-surgical/surgical procedures by physically removing the oral biofilm, dental calculus, and diseased periodontal tissues, and using adjunctive agents. However, these treatment modalities may not always be successful owing to patient(host) and clinical factors. This led to the development of host modulation and local drug delivery in periodontal therapy (77).

Nanosystems show great potential for improving periodontal drug delivery, utilizing various forms such as polymeric nanoparticles, liposomes, exosomes, and nanofibers (78). Their adaptable properties allow for effective targeting and retention in the oral cavity while protecting drugs from pH changes and enzymatic degradation. These structures can be designed for controlled drug release in response to specific conditions in the periodontal environment, addressing three main therapeutic strategies: antibacterial therapy, immunomodulatory therapy, and tissue regeneration. Additionally, the high surface area of nanoparticles allows for significant drug loading, enhancing treatment efficacy through combinations of therapies (79–81).

The role of nanosystems as immunomodulating agents in periodontal therapy can be viewed from the perspective of targeted local delivery for regulating the immune responses, and interactive activity influencing antimicrobial and tissue regenerative. Current strategies utilizing nanoparticle-based approaches for immune regulation, antibacterial treatment, and periodontal tissue regeneration have been reported in the literature (82, 83).

The following subsections examine nanosystems as immunomodulators and as synergistic agents in periodontal therapy.

### 4.1 Nanosystem-mediated immunomodulation

Nanosystems can be used as carriers for conventional drugs (like antibiotics or anti-inflammatory agents), and biologic agents (like cytokines, antibodies). These nanosystems can offer targeted and controlled release, improving the efficacy of these agents and reducing side effects. Nanosystems can encapsulate antibiotics, anti-inflammatory drugs, or even molecules to deliver them to the site of interest more efficiently. These systems ensure that the agents are released in a controlled manner, increasing their local concentration and effectiveness while minimizing systemic side effects.

Modulating cytokine secretion is essential for restoring immune balance in periodontitis treatment. A baicalin- and baicalein-loaded mesoporous silica nanoparticles (Nano-BA and Nano-BE) was developed to regulate inflammatory cytokine secretion (84). In an inflammation cell model using primary human gingival epithelial cells stimulated with IL-1β, these nanoparticles were effective in downregulating cytokines that contributed to inflammation, such as epithelial cell-derived neutrophil-activating peptide, monocyte chemoattractant protein-1, and IL-8. Another study (85) utilized polydopamine nanoparticles, synthesized through self-polymerization. These nanoparticles significantly reduced levels of pro-inflammatory mediators TNF-α and IL-1β in mice. Following treatment with polydopamine nanoparticles, serum cytokine levels normalized, along with liver enzyme levels. Additionally, polydopamine nanoparticles effectively decreased ROS levels in lipopolysaccharide (LPS)-induced inflammation, suggesting that lowering ROS may have helped to regulate inflammatory cytokine levels during periodontitis treatment.

One animal model study of periodontitis showed that resveratrol nanoparticles could decrease levels of pro-inflammatory cytokines, increase levels of anti-inflammatory cytokines, which resulted in a significant therapeutic effect on periodontal inflammation (86).

Biologically derived material nanosystems have been reported to be efficient in macrophage modulation and restoring T-helper cell 17(Th17)/Treg balance, with an imbalance leading to inflammation and tissue damage.

Exosomes are nanosized vesicles secreted by cells that can modulate macrophage phenotypes. Dental pulp stem cell-derived exosomes (DPSCs-Exo) combined with chitosan hydrogels were used to treat periodontitis in mice (87). The treatment significantly increased anti-inflammatory markers and reduced pro-inflammatory markers in macrophages, suggesting that DPSCs-Exo can facilitate macrophage polarization toward an anti-inflammatory state. Exosomes from periodontal membrane stem cells (PDLSC-exo) could correct the Th17/Treg imbalance in periodontitis patients (88). The exosomes influenced the expression of transcription factors related to these cell types and transferred microRNA(miR)-155-5p, which helped regulate histone deacetylase protein levels in CD4+ T cells. Exosomes from mesenchymal stem cells (3D-exo) showed a reduction in Th17 cells and an increase in Tregs in a periodontitis mouse model (89).

Liposomes loaded with resveratrol (Lipo-RSV) were developed to shift macrophages from the M1(pro-inflammatory) to M2 (anti-inflammatory) phenotype (90). This treatment increased M2 markers (for example, CD206) and decreased M1 markers (for example, CD86), indicating a successful polarization shift. The mechanism involved the inhibition of signal transducer and activator of transcription (STAT)1 phosphorylation and promotion of STAT3 phosphorylation, leading to reduced pro-inflammatory cytokines and increased IL-10 levels.

Cerium oxide (CeO2) acts as a nanoenzyme that scavenges ROS, which can exacerbate inflammation if produced excessively. Wang et al. (80), constructed a nanocomplex combining CeO2 with quercetin to enhance ROS scavenging. This inhibited M1 polarization and promoted M2 polarization, downregulated pro-inflammatory cytokines, and upregulated anti-inflammatory cytokines in an animal model with periodontal inflammation. It was found to be effective in scavenging ROS and converting pro-inflammatory macrophages to the anti-inflammatory type removing inflammation.

Treatment with this nanocomplex led to a significant increase in M2 macrophages and decreased pro-inflammatory markers, indicating its potential for regulating the immune environment.

### 4.2 Synergistic role of nanosystems

To explore the potential beneficial effect of immunomodulatory nanosystems additional specific therapeutic agents have been combined, such as antimicrobials, and mediators to regenerate the lost periodontal tissues. Several interesting studies have provided information for considering such synergistic approaches.

Dong and co-workers (91), developed a hydrogel modified with gold nanoparticles and epigallocatechin gallate for combined antibacterial and periodontal regeneration therapy. This hybrid demonstrated effective photothermal effects, raising temperatures to 50.7°C under 808 nm near-infrared laser irradiation. It achieved antibacterial rates of 92% against *Escherichia coli* and 94% against *Staphylococcus aureus*, causing significant damage to the bacterial cell membranes. Additionally, treatment with this formulation enhanced alkaline phosphatase (ALP) activity five-fold and increased calcified nodule formation three-fold in bone marrow stem cells (BMSCs). The mRNA expression levels of ALP, runt-related transcription factor 2 (RUNX2), and osteocalcin (OCN) also increased, likely due to the sustained release of epigallocatechin gallate triggered by near-infrared light. Compared to the control, this treatment resulted in closer alveolar bone crest to cementoenamel junction distances and better-organized collagen fibers. A dual pH- and enzyme-responsive nanosystem (DSPE-PEG-PAMAM/ALA/Mino) for trimodal synergistic treatment was investigated (78). This system combined two key components: poly(aminoamine) (PAMAM), which was loaded with the antimicrobial minocycline hydrochloride and responded to pH changes, and 1,2-distearoyl-sn-glycero-3-phosphoethanolamine-polyethylene glycol (DSPE-PEG), which carried the antioxidant alpha lipoic acid (ALA) and responded to bacterial enzymes. Under periodontitis conditions, this system reasonably released both minocycline and ALA. Pharmacodynamic studies showed that treatment with DSPE-PEG-PAMAM/ALA/Mino resulted in minimal bacterial presence, indicating strong antibacterial activity. Additionally, it inhibited inflammation by reducing ROS and inducible nitric oxide synthase levels. The treatment also significantly decreased the alveolar bone crest to cementoenamel junction distances by an average of 0.357 mm, demonstrating its effectiveness in periodontal repair. Mechanistic studies revealed that the system upregulated mRNA expression of ALP and OCN, promoting osteogenic differentiation in BMSCs. This nanosystem effectively combines antibacterial, immunomodulatory, and periodontal repair functions for treating periodontitis.

Nanosystems are potentially helpful in enhancing the retention time of local drug concentrations in the periodontal tissue sites. Smart hydrogel platforms for periodontitis therapy have been reported (92). These hydrogels feature responsive groups allowing *in situ* phase transitions between solution and solid states within the periodontal pocket. An injectable photosensitive hydrogel incorporating dexamethasone-loaded zeolitic imidazolate framework-8 (ZIF-8) nanoparticles into a photo-crosslinked matrix, to enhance treatment efficacy was developed. Additionally, these smart hydrogels could control the release of nanoparticles in response to stimuli such as light, pH, enzymes, and ROS. One study also showed that a chitosan/sodium β-glycerophosphate hydrogel exhibited pH-responsive release properties, collapsing significantly at pH 4.0, which is typical of acidic periodontal environments (93). One research group created bio-sponge materials from carboxymethyl chitosan, poly-gamma-glutamic acid, and platelet-rich plasma, which promoted blood clotting by releasing epidermal growth factor and vascular endothelial growth factor (94). In periodontitis treatment, such absorbable bio-sponge platforms support bone regeneration. For instance, collagen sponges loaded with mesenchymal stem cell exosomes enhanced the migration and proliferation of periodontal ligament cells, facilitating periodontal regeneration.

A nanosystem of chitosan nanoparticles embedded in polylactic acid nanofibers (CS/PLA) has been shown to enhance the osteogenic differentiation of BMSCs and improve extracellular matrix mineralization (95). The inclusion of chitosan nanoparticles increased the mechanical strength of the fibers while preserving space for regeneration. The CS/PLA scaffold provided structural cues that guided cellular alignment, beneficial for tissue regeneration, particularly in the periodontal ligament. Alizarin red staining indicated that these nanofibers could promote the formation of mineralized nodules by BMSCs and upregulated osteoblast-related mRNA factors like RUNX2 and osteoprotegerin (OPG). Similar effects were observed with PLA/calcium alginate (PLA/CA) nanofibers (96), suggesting that using polysaccharide composites with PLA nanofibers could be an effective strategy for treating periodontitis. AMG-487 [an 8-azaquinazolinone which is a chemokine [C-X-C motif] receptor 3[ CXCR3] antagonist]nanoparticles, were incorporated into liposome nanoparticles made from palmitic acid and cholesterol to disrupt osteoclastogenesis (97). Tartrate-resistant acid phosphatase (TRAP) staining indicated that the treatment with AMG-487 nanoparticles -loaded liposomes successfully inhibited osteoclast formation. Additionally, micro-CT analysis revealed a 27.8% reduction in bone loss after one week of treatment. This suggests that CXCR3 blockers could be a promising target for addressing bone loss in periodontitis by inhibiting osteoclastogenesis.

The relevant nanosystems and their effect on periodontal therapy are summarized in Table 1.

**Table 1:** Nanosystems and their immunomodulatory effects on periodontal therapy.

Nanosystem	Result	Reference
Nanoenzyme CeO2 with Quercetin	Decreased M1-, increased M2-macrophages, scavenging of ROS, reduction in inflammation	Wang et al., (80)
Mesoporous silica nanoparticles with Baicalin and Baicalein	Decreased IL-1β-triggered pro-inflammatory cytokines such as MCP-1 and IL-8, reduction in inflammation	Liu et al., (84)
Polydopamine nanoparticles	Reduced levels of pro-inflammatory mediators TNF-α and IL-1β, decreased ROS, reduction in inflammation	Bao et al., (85)
DPSC Exosomes	Converted M1-to M2-macrophages, reduction in Th17 cells and an increase in Tregs, reduction in inflammation	Shen et al., (87)
Liposome with Resveratrol	Converted M1-to M2-macrophages, reduction in inflammation	Shi et al., (90)
Gold nanoparticles and epigallocatechin gallate	ALP, RUNX2, and OCN increased, periodontal regeneration	Dong et al., (91)
DSPE-PEG-PAMAM/ALA/Mino	Reduced ROS and nitric oxide synthase, reduction in inflammation; ALP and OCN increased, promoting osteogenesis	Wang et al., (78)
Exosomes (loaded in collagen sponges)	Periodontal regeneration	Tang. et al., (94)
CS/PLA	Osteogenesis periodontal regeneration	Shen et al., (95)
PLA/CA nanofibers	Osteogenesis periodontal regeneration	Ye et al., (96)
Liposome with AMG-487	Osteoclastogenic disruption	Lari et al., (97)

CeO2, cerium oxide; M1 and M2, macrophage phenotypes; IL, Interleukin; MCP, monocyte chemoattractant protein-1; TNF, tumor necrosis factor; ROS, reactive oxygen species; DPSC, dental pulp stem cell; Th, helper T-cell; ALP, alkaline phosphatase; RUNX2, runt-related transcription factor 2; OCN, osteocalcin; DSPE-PEG, 1,2-distearoyl-sn-glycero-3-phosphoethanolamine-polyethylene glycol; PAMAM, poly aminoamine; ALA, alpha lipoic acid; MINO, minocycline; CS, chitosan nanoparticles; PLA, polylactic acid nanofibers; CA, calcium alginate; AMG-487, 8-azaquinazolinone-a chemokine [C-X-C motif] receptor 3[ CXCR3] antagonist.

## 5 Challenges, limitations, and clinical implications

While the potential of immunomodulatory nanosystems in periodontitis is significant, some challenges need to be addressed for clinical application, as most of the evidence is based on animal studies.

Firstly, safety. The long-term safety of nanoparticles needs to be evaluated thoroughly. The potential for toxicity, especially if nanoparticles accumulate in tissues, is an ongoing concern. Second, biocompatibility. Nanoparticles must be designed to interact safely with biological tissues. Surface modifications to improve their biocompatibility and reduce immune system recognition are critical. Third, targeting and specificity. While nanoparticles can be engineered for targeted delivery, achieving precise localization to the periodontal tissues and minimizing off-target effects remains a challenge. Lastly, clinical efficacy. Major research in this area focused on human clinical trials is warranted as the body of evidence is relatively just the beginning.

## 6 Conclusion

The growing understanding of immunological regulation in periodontitis, combined with advancements in nanotechnology is driving the development of nanosystems. Unlike traditional clinical immunomodulatory treatments, which are often palliative, nanosystems target the underlying causes of periodontitis to offer disease resolution. While nanosystems are demonstrating significant promise in preclinical studies, their clinical application still requires thorough evaluation. Continued refinement and innovation in nanosystems strategies are essential, with the expectation that they will soon offer new treatment options for periodontitis.

## References

[1] Jindal A Sarkar S Alam A. Nanomaterials-mediated immunomodulation for cancer therapeutics. Front Chem. (2021) 9:629635. 10.3389/fchem.2021.629635 33708759 PMC7940769

[2] Parkin J Cohen B. An overview of the immune system. Lancet. (2001) 357(9270):1777–89. 10.1016/S0140-6736(00)04904-7 11403834

[3] Hato T Dagher PC. How the innate immune system senses trouble and causes trouble. Clin J Am Soc Nephrol. (2015) 10(8):1459–69. 10.2215/CJN.04680514 25414319 PMC4527020

[4] Netea MG Schlitzer A Placek K Joosten LAB Schultze JL. Innate and adaptive immune memory: an evolutionary continuum in the host’s response to pathogens. Cell Host Microbe. (2019) 25(1):13–26. 10.1016/j.chom.2018.12.006 30629914

[5] Farber DL Netea MG Radbruch A Rajewsky K Zinkernagel RM. Immunological memory: lessons from the past and a look to the future. Nat Rev Immunol. (2016) 16(2):124–8. 10.1038/nri.2016.13 26831526

[6] Eisenbarth SC Baumjohann D Craft J Fazilleau N Ma CS Tangye SG CD4^+^ T cells that help B cells - a proposal for uniform nomenclature. Trends Immunol. (2021) 42(8):658–69. 10.1016/j.it.2021.06.003 34244056 PMC8324560

[7] Dunn GP Bruce AT Ikeda H Old LJ Schreiber RD. Cancer immunoediting: from immunosurveillance to tumor escape. Nat Immunol. (2002) 3(11):991–8. 10.1038/ni1102-991 12407406

[8] Finlay BB McFadden G. Anti-immunology: evasion of the host immune system by bacterial and viral pathogens. Cell. (2006) 124(4):767–82. 10.1016/j.cell.2006.01.034 16497587

[9] Feng X Xu W Li Z Song W Ding J Chen X. Immunomodulatory nanosystems. Adv Sci (Weinh). (2019) 6(17):1900101. 10.1002/advs.201900101 31508270 PMC6724480

[10] van der Burg SH Arens R Ossendorp F van Hall T Melief CJ. Vaccines for established cancer: overcoming the challenges posed by immune evasion. Nat Rev Cancer. (2016) 16(4):219–33. 10.1038/nrc.2016.16 26965076

[11] Wang H Mooney DJ. Biomaterial-assisted targeted modulation of immune cells in cancer treatment. Nat Mater. (2018) 17(9):761–72. 10.1038/s41563-018-0147-9 30104668

[12] Ahamad N Kar A Mehta S Dewani M Ravichandran V Bhardwaj P Immunomodulatory nanosystems for treating inflammatory diseases. Biomaterials. (2021) 274:120875. 10.1016/j.biomaterials.2021.120875 34010755

[13] Khatun S Putta CL Hak A Rengan AK. Immunomodulatory nanosystems: an emerging strategy to combat viral infections. Biomater Biosyst. (2023) 9:100073. 10.1016/j.bbiosy.2023.100073 36967725 PMC10036237

[14] Gordon JR Ma Y Churchman L Gordon SA Dawicki W. Regulatory dendritic cells for immunotherapy in immunologic diseases. Front Immunol. (2014) 5(7):1–19. 10.3389/fimmu.2014.00007 24550907 PMC3907717

[15] Shen Y Hao T Ou S Hu C Chen L. Applications and perspectives of nanomaterials in novel vaccine development. Medchemcomm. (2017) 9(2):226–38. 10.1039/C7MD00158D 30108916 PMC6083789

[16] Huaux F. Emerging role of immunosuppression in diseases induced by micro- and nano-particles: time to revisit the exclusive inflammatory scenario. Front Immunol. (2018) 9:2364. 10.3389/fimmu.2018.02364 30510551 PMC6252316

[17] Cifuentes-Rius A Desai A Yuen D Johnston APR Voelcker NH. Inducing immune tolerance with dendritic cell-targeting nanomedicines. Nat Nanotechnol. (2021) 16(1):37–46. 10.1038/s41565-020-00810-2 33349685

[18] Batool F Özçelik H Stutz C Gegout PY Benkirane-Jessel N Petit C Modulation of immune-inflammatory responses through surface modifications of biomaterials to promote bone healing and regeneration. J Tissue Eng. (2021) 12:20417314211041428. 10.1177/20417314211041428 34721831 PMC8554547

[19] Papapanou PN Sanz M Buduneli N Dietrich T Feres M Fine DH Periodontitis: consensus report of workgroup 2 of the 2017 world workshop on the classification of periodontal and peri-implant diseases and conditions. J Periodontol. (2018) 89(Suppl 1):S173–82. 10.1002/JPER.17-0721 29926951

[20] Eke PI Wei L Borgnakke WS Thornton-Evans G Zhang X Lu H Periodontitis prevalence in adults ≥ 65 years of age, in the USA. Periodontol 2000. (2016) 72(1):76–95. 10.1111/prd.12145 27501492 PMC8223257

[21] Jiao J Jing W Si Y Feng X Tai B Hu D The prevalence and severity of periodontal disease in mainland China: data from the fourth national oral health survey (2015-2016). J Clin Periodontol. (2021) 48(2):168–79. 10.1111/jcpe.13396 33103285

[22] Hajishengallis G. Interconnection of periodontal disease and comorbidities: evidence, mechanisms, and implications. Periodontol 2000. (2022) 89(1):9–18. 10.1111/prd.12430 35244969 PMC9018559

[23] Grossi SG Zambon JJ Ho AW Koch G Dunford RG Machtei EE Assessment of risk for periodontal disease. I. Risk indicators for attachment loss. J Periodontol. (1994) 65(3):260–7. 10.1902/jop.1994.65.3.260 8164120

[24] Lang NP. Commentary: bacteria play a critical role in the etiology of periodontal disease. J Periodontol. (2014) 85(2):211–3. 10.1902/jop.2013.130699 24479635

[25] Meyle J Chapple I. Molecular aspects of the pathogenesis of periodontitis. Periodontol 2000. (2015) 69(1):7–17. 10.1111/prd.12104 26252398

[26] Van Dyke TE Kornman KS. Inflammation and factors that may regulate inflammatory response. J Periodontol. (2008) 79(8 Suppl):1503–7. 10.1902/jop.2008.080239 18673003

[27] Hajishengallis G. Periodontitis: from microbial immune subversion to systemic inflammation. Nat Rev Immunol. (2015) 15(1):30–44. 10.1038/nri3785 25534621 PMC4276050

[28] Medzhitov R. Inflammation 2010: new adventures of an old flame. Cell. (2010) 140(6):771–6. 10.1016/j.cell.2010.03.006 20303867

[29] Ebersole JL Singer RE Steffensen B Filloon T Kornman KS. Inflammatory mediators and immunoglobulins in GCF from healthy, gingivitis and periodontitis sites. J Periodontal Res. (1993) 28(6 Pt 2):543–6. 10.1111/j.1600-0765.1993.tb02121.x 8263728

[30] Socransky SS Haffajee AD. Periodontal microbial ecology. Periodontol 2000. (2005) 38:135–87. 10.1111/j.1600-0757.2005.00107.x 15853940

[31] Cekici A Kantarci A Hasturk H Van Dyke TE. Inflammatory and immune pathways in the pathogenesis of periodontal disease. Periodontol 2000. (2014) 64(1):57–80. 10.1111/prd.12002 24320956 PMC4500791

[32] Sapna G Gokul S Bagri-Manjrekar K. Matrix metalloproteinases and periodontal diseases. Oral Dis. (2014) 20(6):538–50. 10.1111/odi.12159 23849049

[33] Roberts FA Darveau RP. Microbial protection and virulence in periodontal tissue as a function of polymicrobial communities: symbiosis and dysbiosis. Periodontol 2000. (2015) 69(1):18–27. 10.1111/prd.12087 26252399 PMC4530467

[34] Hajishengallis G Chavakis T Lambris JD. Current understanding of periodontal disease pathogenesis and targets for host-modulation therapy. Periodontol 2000. (2020) 84(1):14–34. 10.1111/prd.12331 32844416 PMC7457922

[35] Drexler KE. Molecular engineering: an approach to the development of general capabilities for molecular manipulation. Proc Natl Acad Sci U S A. (1981) 78(9):5275–8. 10.1073/pnas.78.9.5275 16593078 PMC348724

[36] Zhang WH Hu XX Zhang XB. Dye-doped fluorescent silica nanoparticles for live cell and *in vivo* bioimaging. Nanomaterials (Basel). (2016) 6(5):81. 10.3390/nano6050081 28335209 PMC5302498

[37] Cherukula K Manickavasagam Lekshmi K Uthaman S Cho K Cho CS Park IK. Multifunctional inorganic nanoparticles: recent progress in thermal therapy and imaging. Nanomaterials (Basel). (2016) 6(4):76. 10.3390/nano6040076 28335204 PMC5302572

[38] Petros RA DeSimone JM. Strategies in the design of nanoparticles for therapeutic applications. Nat Rev Drug Discov. (2010) 9(8):615–27. 10.1038/nrd2591 20616808

[39] Duncan R Gaspar R. Nanomedicine(s) under the microscope. Mol Pharm. (2011) 8(6):2101–41. 10.1021/mp200394t 21974749

[40] Bhattacharya R Mukherjee P. Biological properties of “naked” metal nanoparticles. Adv Drug Deliv Rev. (2008) 60(11):1289–306. 10.1016/j.addr.2008.03.013 18501989

[41] Jayaraman P Gandhimathi C Venugopal JR Becker DL Ramakrishna S Srinivasan DK. Controlled release of drugs in electrosprayed nanoparticles for bone tissue engineering. Adv Drug Deliv Rev. (2015) 94:77–95. 10.1016/j.addr.2015.09.007 26415888

[42] Zhang Q Dehaini D Zhang Y Zhou J Chen X Zhang L Neutrophil membrane-coated nanoparticles inhibit synovial inflammation and alleviate joint damage in inflammatory arthritis. Nat Nanotechnol. (2018) 13(12):1182–90. 10.1038/s41565-018-0254-4 30177807

[43] Li R He Y Zhu Y Jiang L Zhang S Qin J Route to rheumatoid arthritis by macrophage-derived microvesicle-coated nanoparticles. Nano Lett. (2019) 19(1):124–34. 10.1021/acs.nanolett.8b03439 30521345

[44] Chen X Liu Y Wen Y Yu Q Liu J Zhao Y A photothermal-triggered nitric oxide nanogenerator combined with siRNA for precise therapy of osteoarthritis by suppressing macrophage inflammation. Nanoscale. (2019) 11(14):6693–709. 10.1039/C8NR10013F 30900717

[45] Schett G Elewaut D McInnes IB Dayer JM Neurath MF. How cytokine networks fuel inflammation: toward a cytokine-based disease taxonomy. Nat Med. (2013) 19(7):822–4. 10.1038/nm.3260 23836224

[46] Medara N Lenzo JC Walsh KA Reynolds EC Darby IB O'Brien-Simpson NM. A review of T helper 17 cell-related cytokines in serum and saliva in periodontitis. Cytokine. (2021) 138:155340. 10.1016/j.cyto.2020.155340 33144024

[47] Berger A. Th1 and Th2 responses: what are they? Br Med J. (2000) 321(7258):424. 10.1136/bmj.321.7258.424 10938051 PMC27457

[48] Wilson DS Dalmasso G Wang L Sitaraman SV Merlin D Murthy N. Orally delivered thioketal nanoparticles loaded with TNF-α-siRNA target inflammation and inhibit gene expression in the intestines. Nat Mater. (2010) 9(11):923–8. 10.1038/nmat2859 20935658 PMC3142359

[49] Schwartz DM Kanno Y Villarino A Ward M Gadina M O’Shea JJ. JAK Inhibition as a therapeutic strategy for immune and inflammatory diseases. Nat Rev Drug Discov. (2017) 16(12):843–62. 10.1038/nrd.2017.201 Erratum in: Nat Rev Drug Discov. 2017 17(1):78. doi: 10.1038/nrd.2017.267. 29104284

[50] Lee Y Kim H Kang S Lee J Park J Jon S. Bilirubin nanoparticles as a nanomedicine for anti-inflammation therapy. Angew Chem Int Ed Engl. (2016) 55(26):7460–3. 10.1002/anie.201602525 27144463

[51] Thamphiwatana S Angsantikul P Escajadillo T Zhang Q Olson J Luk BT Macrophage-like nanoparticles concurrently absorbing endotoxins and proinflammatory cytokines for sepsis management. Proc Natl Acad Sci U S A. (2017) 114(43):11488–93. 10.1073/pnas.1714267114 29073076 PMC5664555

[52] Kolaczkowska E Kubes P. Neutrophil recruitment and function in health and inflammation. Nat Rev Immunol. (2013) 13(3):159–75. 10.1038/nri3399 23435331

[53] Ziraldo C Vodovotz Y Namas RA Almahmoud K Tapias V Mi Q Central role for MCP-1/CCL2 in injury-induced inflammation revealed by *in vitro*, in silico, and clinical studies. PLoS One. (2013) 8(12):e79804. 10.1371/journal.pone.0079804 24312451 PMC3849193

[54] Fromen CA Kelley WJ Fish MB Adili R Noble J Hoenerhoff MJ Neutrophil-particle interactions in blood circulation drive particle clearance and Alter neutrophil responses in acute inflammation. ACS Nano. (2017) 11(11):10797–807. 10.1021/acsnano.7b03190 29028303 PMC5709153

[55] Liu YC Zou XB Chai YF Yao YM. Macrophage polarization in inflammatory diseases. Int J Biol Sci. (2014) 10(5):520–9. 10.7150/ijbs.8879 24910531 PMC4046879

[56] Ponzoni M Pastorino F Di Paolo D Perri P Brignole C. Targeting macrophages as a potential therapeutic intervention: impact on inflammatory diseases and cancer. Int J Mol Sci. (2018) 19(7):1953. 10.3390/ijms19071953 29973487 PMC6073303

[57] Brown BN Ratner BD Goodman SB Amar S Badylak SF. Macrophage polarization: an opportunity for improved outcomes in biomaterials and regenerative medicine. Biomaterials. (2012) 33(15):3792–802. 10.1016/j.biomaterials.2012.02.034 22386919 PMC3727238

[58] Miao X Leng X Zhang Q. The current state of nanoparticle-induced macrophage polarization and reprogramming research. Int J Mol Sci. (2017) 18(2):336. 10.3390/ijms18020336 28178185 PMC5343871

[59] Han J Kim YS Lim MY Kim HY Kong S Kang M Dual roles of graphene oxide to attenuate inflammation and elicit timely polarization of macrophage phenotypes for cardiac repair. ACS Nano. (2018) 12(2):1959–77. 10.1021/acsnano.7b09107 29397689

[60] van der Veeken J Gonzalez AJ Cho H Arvey A Hemmers S Leslie CS Memory of inflammation in regulatory T cells. Cell. (2016) 166(4):977–90. 10.1016/j.cell.2016.07.006 27499023 PMC4996371

[61] Liu Q Wang X Liu X Kumar S Gochman G Ji Y Use of polymeric nanoparticle platform targeting the liver to induce treg-mediated antigen-specific immune tolerance in a pulmonary allergen sensitization model. ACS Nano. (2019) 13(4):4778–94. 10.1021/acsnano.9b01444 30964276 PMC6506187

[62] Thomas TP Goonewardena SN Majoros IJ Kotlyar A Cao Z Leroueil PR Folate-targeted nanoparticles show efficacy in the treatment of inflammatory arthritis. Arthritis Rheum. (2011) 63(9):2671–80. 10.1002/art.30459 21618461 PMC3168725

[63] Ma L Liu TW Wallig MA Dobrucki IT Dobrucki LW Nelson ER Efficient targeting of adipose tissue macrophages in obesity with polysaccharide nanocarriers. ACS Nano. (2016) 10(7):6952–62. 10.1021/acsnano.6b02878 27281538

[64] Beldman TJ Senders ML Alaarg A Pérez-Medina C Tang J Zhao Y Hyaluronan nanoparticles selectively target plaque-associated macrophages and improve plaque stability in atherosclerosis. ACS Nano. (2017) 11(6):5785–99. 10.1021/acsnano.7b01385 28463501 PMC5492212

[65] Li L Guo J Wang Y Xiong X Tao H Li J A broad-spectrum ROS-eliminating material for prevention of inflammation and drug-induced organ toxicity. Adv Sci (Weinh). (2018) 5(10):1800781. 10.1002/advs.201800781 30356945 PMC6193162

[66] Chen Z Vong CT Gao C Chen S Wu X Wang S Bilirubin nanomedicines for the treatment of reactive oxygen species (ROS)-mediated diseases. Mol Pharm. (2020) 17(7):2260–74. 10.1021/acs.molpharmaceut.0c00337 32433886

[67] Liu T Xiao B Xiang F Tan J Chen Z Zhang X Ultrasmall copper-based nanoparticles for reactive oxygen species scavenging and alleviation of inflammation related diseases. Nat Commun. (2020) 11(1):2788. 10.1038/s41467-020-16544-7 32493916 PMC7270130

[68] Li J Jiang X Li H Gelinsky M Gu Z. Tailoring materials for modulation of macrophage fate. Adv Mater. (2021) 33(12):e2004172. 10.1002/adma.202004172 33565154 PMC9245340

[69] Xiong Y Chu X Yu T Knoedler S Schroeter A Lu L Reactive oxygen species-scavenging nanosystems in the treatment of diabetic wounds. Adv Healthc Mater. (2023) 12(25):e2300779. 10.1002/adhm.202300779 37051860

[70] Gao P Wei R Chen Y Li X Pan W Li N Pt nanozyme-bridged covalent organic framework-aptamer nanoplatform for tumor targeted self-strengthening photocatalytic therapy. Biomaterials. (2023) 297(9):122109. 10.1016/j.biomaterials.2023.122109 37058901

[71] Ganugula R Arora M Saini P Guada M Kumar MNVR. Next generation precision-polyesters enabling optimization of ligand-receptor stoichiometry for modular drug delivery. J Am Chem Soc. (2017) 139(21):7203–16. 10.1021/jacs.6b13231 28395139

[72] Wang D Zhang L Wang C Cheng Z Zheng W Xu P Missing-linker-confined single-atomic pt nanozymes for enzymatic theranostics of tumor. Angew Chem Int Ed Engl. (2023) 62(19):e202217995. 10.1002/anie.202217995 36896734

[73] Panigrahy D Gilligan MM Huang S Gartung A Cortés-Puch I Sime PJ Inflammation resolution: a dual-pronged approach to averting cytokine storms in COVID-19? Cancer Metastasis Rev. (2020) 39(2):337–40. 10.1007/s10555-020-09889-4 32385712 PMC7207990

[74] Hu G Guo M Xu J Wu F Fan J Huang Q Nanoparticles targeting macrophages as potential clinical therapeutic agents against cancer and inflammation. Front Immunol. (2019) 10:1998. 10.3389/fimmu.2019.01998 31497026 PMC6712945

[75] Kraynak CA Huang W Bender EC Wang JL Hanafy MS Cui Z Apoptotic body-inspired nanoparticles target macrophages at sites of inflammation to support an anti-inflammatory phenotype shift. Int J Pharm. (2022) 618:121634. 10.1016/j.ijpharm.2022.121634 35247497 PMC9007911

[76] Anselmo AC Mitragotri S. A review of clinical translation of inorganic nanoparticles. AAPS J. (2015) 17(5):1041–54. 10.1208/s12248-015-9780-2 25956384 PMC4540735

[77] Kim WJ Soh Y Heo SM. Recent advances of therapeutic targets for the treatment of periodontal disease. Biomol Ther (Seoul). (2021) 29(3):263–7. 10.4062/biomolther.2021.001 33731493 PMC8094066

[78] Wang L Li Y Ren M Wang X Li L Liu F Ph and lipase-responsive nanocarrier-mediated dual drug delivery system to treat periodontitis in diabetic rats. Bioact Mater. (2022) 18:254–66. 10.1016/j.bioactmat.2022.02.008 35387157 PMC8961308

[79] Tian Y Li Y Liu J Lin Y Jiao J Chen B Photothermal therapy with regulated Nrf2/NF-κB signaling pathway for treating bacteria-induced periodontitis. Bioact Mater. (2022) 9:428–45. 10.1016/j.bioactmat.2021.07.033 34820581 PMC8586811

[80] Wang Y Li C Wan Y Qi M Chen Q Sun Y Quercetin-loaded ceria nanocomposite potentiate dual-directional immunoregulation via macrophage polarization against periodontal inflammation. Small. (2021) 17(41):e2101505. 10.1002/smll.202101505 34499411

[81] Ding Y Wang Y Li J Tang M Chen H Wang G Microemulsion-thermosensitive gel composites as *in situ*-forming drug reservoir for periodontitis tissue repair through alveolar bone and collagen regeneration strategy. Pharm Dev Technol. (2023) 28(1):30–9. 10.1080/10837450.2022.2161574 36541732

[82] Mi G Shi D Wang M Webster TJ. Reducing bacterial infections and biofilm formation using nanoparticles and nanostructured antibacterial surfaces. Adv Healthc Mater. (2018) 7(13):e1800103. 10.1002/adhm.201800103 29790304

[83] Makabenta JMV Nabawy A Li CH Schmidt-Malan S Patel R Rotello VM. Nanomaterial-based therapeutics for antibiotic-resistant bacterial infections. Nat Rev Microbiol. (2021) 19(1):23–36. 10.1038/s41579-020-0420-1 32814862 PMC8559572

[84] Liu X He X Jin D Wu S Wang H Yin M A biodegradable multifunctional nanofibrous membrane for periodontal tissue regeneration. Acta Biomater. (2020) 108:207–22. 10.1016/j.actbio.2020.03.044 32251784

[85] Bao X Zhao J Sun J Hu M Yang X. Polydopamine nanoparticles as efficient scavengers for reactive oxygen species in periodontal disease. ACS Nano. (2018) 12(9):8882–92. 10.1021/acsnano.8b04022 30028940

[86] Huangfu H Du S Zhang H Wang H Zhang Y Yang Z Facile engineering of resveratrol nanoparticles loaded with 20(S)-protopanaxadiol for the treatment of periodontitis by regulating the macrophage phenotype. Nanoscale. (2023) 15(17):7894–908. 10.1039/D2NR06452A 37060139

[87] Shen Z Kuang S Zhang Y Yang M Qin W Shi X Chitosan hydrogel incorporated with dental pulp stem cell-derived exosomes alleviates periodontitis in mice via a macrophage-dependent mechanism. Bioact Mater. (2020) 5(4):1113–26. 10.1016/j.bioactmat.2020.07.002 32743122 PMC7371600

[88] Zheng Y Dong C Yang J Jin Y Zheng W Zhou Q Exosomal microRNA-155-5p from PDLSCs regulated Th17/treg balance by targeting sirtuin-1 in chronic periodontitis. J Cell Physiol. (2019) 234(11):20662–74. 10.1002/jcp.28671 31016751

[89] Zhang Y Chen J Fu H Kuang S He F Zhang M Exosomes derived from 3D-cultured MSCs improve therapeutic effects in periodontitis and experimental colitis and restore the Th17 cell/treg balance in inflamed periodontium. Int J Oral Sci. (2021) 13(1):43. 10.1038/s41368-021-00150-4 34907166 PMC8671433

[90] Shi J Zhang Y Zhang X Chen R Wei J Hou J Remodeling immune microenvironment in periodontitis using resveratrol liposomes as an antibiotic-free therapeutic strategy. J Nanobiotechnology. (2021) 19(1):429. 10.1186/s12951-021-01175-x 34930286 PMC8686397

[91] Dong Z Lin Y Xu S Chang L Zhao X Mei X NIR-triggered tea polyphenol-modified gold nanoparticles-loaded hydrogel treats periodontitis by inhibiting bacteria and inducing bone regeneration. Mater Des. (2023) 225:111487. 10.1016/j.matdes.2022.111487

[92] Liu Y Li T Sun M Cheng Z Jia W Jiao K ZIF-8 modified multifunctional injectable photopolymerizable GelMA hydrogel for the treatment of periodontitis. Acta Biomater. (2022) 146:37–48. 10.1016/j.actbio.2022.03.046 35364317

[93] Chen J Zhang X Huang C Cai H Hu S Wan Q Osteogenic activity and antibacterial effect of porous titanium modified with metal-organic framework films. J Biomed Mater Res A. (2017) 105(3):834–46. 10.1002/jbm.a.35960 27885785

[94] Tang J Yi W Yan J Chen Z Fan H Zaldivar-Silva D Highly absorbent bio-sponge based on carboxymethyl chitosan/poly-γ-glutamic acid/platelet-rich plasma for hemostasis and wound healing. Int J Biol Macromol. (2023) 247:125754. 10.1016/j.ijbiomac.2023.125754 37429345

[95] Shen R Xu W Xue Y Chen L Ye H Zhong E The use of chitosan/PLA nano-fibers by emulsion eletrospinning for periodontal tissue engineering. Artif Cells Nanomed Biotechnol. (2018) 46(sup2):419–30. 10.1080/21691401.2018.1458233 29661034

[96] Ye Z Xu W Shen R Yan Y. Emulsion electrospun PLA/calcium alginate nanofibers for periodontal tissue engineering. J Biomater Appl. (2020) 34(6):763–77. 10.1177/0885328219873561 31506032

[97] Lari S Hiyari S de Araújo Silva DN de Brito Bezerra B Ishii M Monajemzadeh S Local delivery of a CXCR3 antagonist decreases the progression of bone resorption induced by LPS injection in a murine model. Clin Oral Investig. (2022) 26(8):5163–9. 10.1007/s00784-022-04484-z 35462591 PMC9710470

